# Acknowledgment to the Reviewers of *Antibodies* in 2022

**DOI:** 10.3390/antib12010008

**Published:** 2023-01-17

**Authors:** Antibodies Editorial Office

**Affiliations:** MDPI AG, St. Alban-Anlage 66, 4052 Basel, Switzerland

High-quality academic publishing is built on rigorous peer review. *Antibodies* was able to uphold its high standards for published papers due to the outstanding efforts of our reviewers. Thanks to the efforts of our reviewers in 2022, the median time to first decision was 21 days and the median time to publication was 51 days. Regardless of whether the articles they examined were ultimately published, the editors would like to express their appreciation and thank the following reviewers for the time and dedication that they have shown Journal:
A.J. RosenspireKenneth A. AndreoniAdam BarbKenta HarayaAdam WisnewskiKevin J. SelvaAfua Adjeiwaa MensahKevin M CoombsAlan SchmaljohnKira V. VyatkinaAlastair LawsonKisung KoAlexey TeplyakovKRISHNA CHAITANYAAli R. JazirehiKristine M. KimAlireza ZarebidokiKumiko Nakada-TsukuiAlvise SernicolaLaura TiberioAmita Datta-MannanLaureline BerthelotAmy XuLong Thanh PhamAndrea L. J. MarschallLuca VaraniAnisuz ZamanLuigi R. CeciAnthony CheungLuis Alvarez-VallinaAnton SholukhM. Quadir SiddiquiArtur ZalevskyMarc HilhorstAtheer ZgairMarco PalmaAvneesh GautamMaria RodriguezBernardo VegaMarie-Alix PoulBingchun ZhaoMark S. CraggBrian D. AdamsMartina JonesBruce WalcheckMatthias ScheweCarlo Cosimo CampaMehdi Arbabi GhahroudiCaterina Maria GambinoMichael G. WellerCharles DumontetMichael KundiChinh Tran-To SuMohamed FodaChiuan LeowMonika Christine Brunner-WeinzierlChristiane MoogNeil GreenspanCiesla MaciejNicolas SabarthColin H. QuinnOhad MazorCzeslaw RadziejewskiOlga BorgesDaisuke TsurutaParamita ChakrabortyDario GenovesePaulina ŻarnowiecDelphine Le RouxPenelope M. DrakeDeng-Ho YangPeter KorstenDenise ToscaniPéter OláhDennis R. GouletPetros ChristopoulosDiana Panayotova-DimitrovaPrabakaran PonrajDiego MoricoliQun ZhouDominique LesuisseRaffaele FrazziDoreen M. FlossRahul MannanE. F. KolesanovaRenata Von Glehn PonsirenasEdwin J. Vazquez-CintronRicardo GiordanoElena CsernokRitthideach YorsaengElena SavvateevaRonald P. TaylorElizabeth Helen KempRoni NugrahaElizabeth J. PriceRosa Rodríguez-PérezEmilia BiałopiotrowiczSafder GanaieEmilia CirilloSaketh ChemuruEmmanuel VictorSanjay DeyErika OrbanScott WetzelErwan MortierSebastian YuFaraz A. ChoudhurySebastien CalboFeliciana Real-FernándezSelami Aykut TemizFlavio Di PisaSeshi SompuramFloris L. Van DelftSharath MadasuFrank BreitlingShin-Ichi OsadaFrank KrauseSina TaefehshokrFrederick J. KaskelSrinath ThirumalairajanGábor KonczSukmook LeeGalia Ramírez-TolozaSyed AhmedGarima ArvikarTae Hyun KangGestur VidarssonTakahiro AnzaiGiorgia ManniTaku KouroGonca E. KarahanTakuya TadaGouveia Zelia LiciniaTaru S DuttHeather BaxTarun KeswaniHyosang BaeTheam Soon LimHyunbo ShimTimm AmendtItai BenharUrszula LisieckaItzel Amaro-EstradaV. Ashutosh RaoIzabela ChmielewskaVeera Venkat Shivaji Rama Rao EdupugantiJamshid TanhaVictoria PolonisJeremy SchmitVladimira BoyadzievaJerome E. TannerWakiro SatoJianlong LouWanessa C. LimaJingzhong GuoWei SunJohn De KruifWibke BayerJosé Germano De SousaWilliam David TolbertJuan C. AlmagroXavier Fulladosa OliverasJulián Esteban Barahona-CorreaXingyi JiangJulius GrzeschikYinzhi LangJurgen SanesYoshiyuki NakamuraKarina Kubiak-OssowskaYutaka MatsudaKasturi BanerjeeZahra RattrayKatharine M. LodgeŽivka Dika


